# P-35. Influenza Hospitalizations among Adults aged 18-64 Years and the Potential Benefit of Recombinant Vaccines: USA, 2012-13 through 2022-23

**DOI:** 10.1093/ofid/ofae631.242

**Published:** 2025-01-29

**Authors:** Laurence Torcel-Pagnon, Laurent Coudeville, Sandra S Chaves

**Affiliations:** Sanofi, LYON, Rhone-Alpes, France; Sanofi, LYON, Rhone-Alpes, France; Sanofi Pasteur, Lyon, Languedoc-Roussillon, France

## Abstract

**Background:**

Influenza can cause severe illness across all age groups. Despite the universal influenza vaccine recommendation in the USA since 2010, vaccination rates among adults aged 18-64 remain low compared to children and the elderly. Given the prevalence of chronic medical conditions (CMC) that heighten the risk of severe outcomes in this population, we aim to document influenza hospitalizations among US adults aged 18-64 and establish the number of additional cases that could be averted by replacing standard dose (SD) with recombinant influenza vaccine (RIV).

Additional averted hospitalizations among adults aged 18-64 when using recombinant influenza vaccine, by varying relative vaccine effectiveness estimates*

**Figure:**
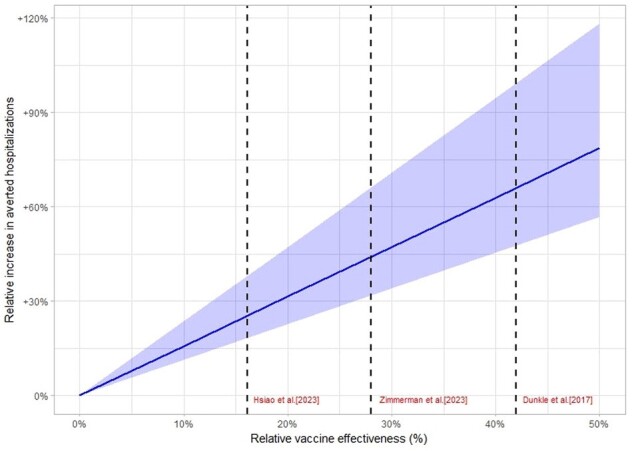

*. Dashed line represents point estimates of relative vaccine effectiveness reported in the literature comparing recombinant influenza vaccine vs. standard dose vaccine. Shaded area around the line represents 95% Confidence Interval. Based on CDC vaccine effectiveness estimates, similar absolute vaccine effectiveness was assumed for both groups (18-49 and 50-64 years).

Dunkle LM, Izikson R, Patriarca P, Goldenthal KL, Muse D, Callahan J, et al. Efficacy of Recombinant Influenza Vaccine in Adults 50 Years of Age or Older. N Engl J Med. 2017;376:2427–36.

Hsiao A, Yee A, Fireman B, Hansen J, Lewis N, Klein NP. Recombinant or Standard-Dose Influenza Vaccine in Adults under 65 Years of Age. N Engl J Med. 2023;389:2245–55.

Zimmerman RK, Nowalk MP, Dauer K, Clarke L, Raviotta JM, Balasubramani GK. Vaccine effective-ness of recombinant and standard dose influenza vaccines against influenza related hospitalization using a retrospective test-negative design. Vaccine. 2023;41:5134–40.

**Methods:**

We used data from the US Centers for Disease Control and Prevention (CDC) on influenza-related hospitalizations from 2012-13 through 2022-23 (excluding 2020-21 season due to lack of influenza virus circulation) and evaluated pooled absolute vaccine effectiveness (aVE). We estimated influenza hospitalizations for those with CMC aged 18-49, applying US CMC prevalence rates from literature. For those aged 50-64, hospitalization estimates were based on CDC data, considering the entire group at-risk due to age and high prevalence of CMC. Using a decision-tree model with varying relative VE (rVE), we assessed the additional benefit of using RIV instead of SD in averting influenza-related hospitalizations among adults aged 18-64.

**Results:**

Over the past decade, the CDC has reported annual influenza related hospitalizations ranging from 37,000 to 204,000 among those aged 18-64. This age group contributed from 21% to 47% of all-age hospitalizations during this period, with higher percentages observed during seasons dominated by H1N1pdm09. Average annual hospitalization rates per 100,000 population were 123.5 for those aged 50-64, and 82.2 for those aged 18-49 with CMC. The adoption of RIV for adults aged 18-64 could prevent additional influenza-related hospitalizations, with the magnitude of benefit depending on the rVE for the season (e.g. 47% [95% Confidence interval 34-71] averted influenza hospitalizations based upon 30% rVE, Figure).

**Conclusion:**

The impact of influenza hospitalizations among adults aged 18-64 cannot be understated. The use of RIV or other enhanced influenza vaccine for non-elderly high-risk groups should be considered. Further research is warranted.

**Disclosures:**

**Laurence Torcel-Pagnon, Msc**, Sanofi: Employee|Sanofi: Stocks/Bonds (Private Company) **Laurent Coudeville, phD**, Sanofi: Employee|Sanofi: Stocks/Bonds (Private Company) **Sandra S. Chaves, MD, MSc**, Sanofi: Employee|Sanofi: Employee|Sanofi: Stocks/Bonds (Private Company)|Sanofi: Stocks/Bonds (Private Company)

